# The measurement of dietary species richness reveals that a higher consumption of dietary fibre, fish, fruits and vegetables, is associated with greater food biodiversity in UK diets

**DOI:** 10.1017/S1368980025000473

**Published:** 2025-04-11

**Authors:** Magaly Aceves-Martins, Anneli Löfstedt, Carlos Francisco Moreno-García, Elizabeth H Zandstra, Anne J Wanders, Baukje de Roos

**Affiliations:** 1 Rowett Institute, University of Aberdeen, Aberdeen, UK; 2 School of Computing, Engineering and Technology, Robert Gordon University, Aberdeen, UK; 3 Unilever Foods Innovation Centre Wageningen, Wageningen, The Netherlands; 4 Wageningen University and Research, Wageningen, The Netherlands

**Keywords:** Food biodiversity, Dietary species richness, Diet quality, Food composition, NDNS

## Abstract

**Objective:**

We determined whether dietary species richness (DSR) (i) can be robustly measured using 4-day food intake data, (ii) is dependent on socio-demographic characteristics and (iii) is associated with diet quality.

**Design:**

The National Diet and Nutrition Survey (NDNS) nutrient databank 2018–2019 was expanded to include FoodEx2 food classifications, ingredients, the number and identity of unique species, Nutrient Rich Food 8·3 (NRF 8·3) Index scores and greenhouse gas emissions. Four-day food intake data and socio-demographic variables were used to calculate diet quality and DSR on the food and diet level.

**Setting:**

The United Kingdom (UK).

**Participants:**

Participants from NDNS 9–11 (2016–2019).

**Results:**

Composite dishes had the highest DSR (median 8 (Q1 = 4, Q3 = 12)), followed by seasoning, sauces and condiments (median 7, (Q1 = 4, Q3 = 10)) and, grains and grain-based products (median 5, (Q1 = 2, Q3 = 7)). Median DSR over 4 days was 49 (Q1 = 43, Q3 = 56; range 14–92), with the first 2 days achieving 80 % of DSR measured over 4 days. DSR was significantly higher in those who were younger, those with a higher household income or those with a lower level of deprivation (all *P* < 0·001). Higher DSR was associated with a small but significant improvement in nutritional quality (*P* < 0·001). Also, adherence to dietary guidelines such as fibre, fruits and vegetables and fish was associated with significantly higher DSR (all *P* < 0·001).

**Conclusions:**

We successfully established DSR based on 4-day food intake data. We also identified opportunities to improve DSR by increasing the consumption of fruits, vegetables, fibre and fish.

Diet diversity is a key element of healthy diets. A wide variety of foods, between and within food groups, is associated with an increased intake of essential nutrients and bioactive components, helping to meet micronutrient requirements and a lowered risk of mortality and diet-related non-communicable diseases^(1)^. Typically, dietary diversity has been calculated by counting food groups consumed over a given period and used as a measure of diet quality in especially low and middle-income countries^(2)^. However, food group scores do not necessarily capture the variability in species and nutrients across diets, and more recently, the concept of food biodiversity has been introduced to measure the diversity of plants, animals and other organisms (e.g. fungi, insects) used for food^(3–5)^. Food biodiversity has already been associated with total and cause-specific mortality across European countries^(5)^, and it is also associated with planetary health: food species biodiversity reduces pressures on single species and supports food and nutrition security in the face of anthropogenic challenges^(6)^. Agriculture is being singled out as a direct threat to 86 % of species facing extinction, primarily due to land conversion, particularly for industrial mono-crop and animal agriculture^(7)^. Estimates are that we have access to 300 000 edible plant species, but that around half of the dietary calories we consume globally are met by only four crops: rice, potatoes, wheat and maize, whilst these are beef, wheat, pork and potato in Europe^(8)^. Consequently, current food systems accelerate biodiversity loss and malnutrition^(9)^.

The measurement of food biodiversity can be divided into three main components: richness, evenness and disparity^(4)^. Richness considers the total number of distinct edible species consumed over a specific period^(4)^, evenness considers the number of unique species and the evenness of their quantities in a diet, measured as the probability that two randomly selected food items belong to the same species^(10)^ and disparity measures the ‘entropy of disorder’ or the differences in the functional traits or ecological roles of the species in the diet^(11)^. All of these indices of diversity measure different features of a diet. Dietary species richness (DSR) has been highlighted as a novel, comprehensive and simple metric for the simultaneous measurement of food biodiversity between and within food groups and the nutritional quality of human diets, capturing both agricultural and wild food biodiversity^(12)^. Recently, DSR was inversely associated with total and cause-specific mortality within the European Prospective Investigation into Cancer and Nutrition (EPIC) cohort. This effect was independent of socio-demographic, lifestyle and other known dietary risk factors^(5)^. Such findings advocate for food biodiversity to be included in public health strategies as a new metric for healthy and sustainable foods and diets, linking the fields of ecological, agricultural and food biodiversity with human and planetary health outcomes. However, how DSR can be best measured, the exact relationship between DSR and diet quality, and what DSR score is required for optimal health, are currently unknown. Such information is required to explore meaningful opportunities for increasing food biodiversity in our diets.

Using National Diet and Nutrition Survey (NDNS) data, we quantified food biodiversity for foods and diets consumed in the UK using the DSR metric. We assessed (i) whether a 4-day food diary is appropriate to capture DSR, (ii) whether there are variations in DSR over 4 days for different consumer segments based on sex, age group, BMI, ethnicity, Index of Multiple Deprivation (IMD), household income and adherence to different dietary guidelines and (iii) whether DSR is associated with diet quality.

## Methods

### Database development

An expanded version of the NDNS nutrient databank 2018–2019 was used in this analysis. This databank included nutritional composition, level of processing (NOVA categories)^(13)^, greenhouse gas (GHG) emissions (farm-to-fork)^(14,15)^ and current price (September 2023 retail prices without adjusting for inflation), for nearly 6000 commonly consumed foods and drinks in the UK^(16–19)^. The Nutrient Rich Food 8·3 (NRF8·3) index scores were also calculated for all NDNS nutrient databank items on a per 418 kJ (100 kcal) basis^(20,21)^. NRF index scores are diet quality indices based on the nutrient density of each food item, accounting for beneficial nutrients, nutrients to limit or a combination of both^(17)^. In addition, each item was categorised according to the European Food Safety Authority’s (EFSA) FoodEx2 food classification, which is a comprehensive food classification and description system designed to standardise how food is described and classified across different food safety domains^(22)^. To calculate DSR, the ingredients for each food and drink item, including composite dishes, were identified using online data from a single major UK retailer^(23)^. Ingredients for homemade dishes were obtained from the BBC Good Food website^(24)^. In the first instance, the list of unique species was taken from the food biodiversity codes assigned to the EPIC cohort’s food list^(5)^ and expanded with twenty-one additional species, resulting in 269 unique species (see online supplementary material, Supplementary Table 1). From this, 216 unique species were found to be present in the foods and drinks of the NDNS nutrient databank. Generic species names and synonyms were produced for each of these species. We then matched the list of species and synonyms to the ingredients list of the NDNS nutrient databank using an R algorithm whilst manually checking for inconsistencies. This approach yielded the total number and type of unique species (i.e. DSR) for each of the >6000 food or drink items in the NDNS nutrient databank. When considering the total list of 216 unique species for the UK database, the highest proportion was provided by fish, fish products and any other marine and freshwater food products (29 %), fruit (24 %) and vegetable species (22 %).

### NDNS analysis

Data were obtained from the annual rolling cross-sectional UK National Diet and Nutrition Survey (NDNS) waves 9–11, which comprise data gathered between 2016–2017 and 2018–2019^(25)^. A 4-day estimated food diary was used, where participants were asked to keep a record of foods and drinks consumed over four consecutive days^(25)^. Only data from participants who completed 3 or 4 d were included in this analysis. We used socio-demographic data on age, sex, BMI, ethnicity, household income and IMD, a widely used metric to classify the relative deprivation of small areas in the UK^(26)^, which are provided in the NDNS. In addition, in the absence of a validated healthy diet index for UK Diets, we used NRF 8·3 index scores estimated at an individual level as a proxy of diet quality. To estimate adherence to dietary guidelines, each participant was categorised as adhering (or not) to the following guidelines: consuming less than 11 % of total energy from saturated fats^(27)^, less than 5 % of total energy from free sugars^(28)^, less than 70 g of red meat per day^(29)^, less than 6 g of salt per day^(30)^, more than 30 g of fibre per day^(28)^, more than 400 g of fruits and vegetables per day^(31)^ or more than 280 g of fish a week^(32)^. Moreover, it was estimated that ultra-processed foods account for almost 60 % of total energy intake in UK diets^(33)^. Based on this, we categorised those consuming ≥60 % of total energy from processed or ultra-processed foods as ‘high processed food eaters’.

### Statistical analysis

For the food-level analysis, Shapiro–Wilk tests were conducted to check for the normality of data in the NDNS nutrient databank. Because of non-normality, median values were used to plot the distributions. Correlations between DSR and different food characteristics (e.g. energy density, GHG emissions and current price) were evaluated using Spearman correlations and confidence intervals (95 %), adjusted for multiple testing using a Bonferroni method.

For the individual diet-level analysis, to estimate the DSR, we considered the absolute number of unique biological species consumed per day per person, across foods and drinks and across the one, two, three and four food diary days. We included spices, extracts and flavourings in foods, considering they are common in composite dishes and that bioactives can have benefits to health even if consumed in small amounts^(34,35)^. However, we excluded extracts and flavourings coded or presented in an unknown nomenclature. Differences in DSR across 4 d and between socio-demographic characteristics were tested with Kruskal–Wallis tests and adjusted for multiple testing using a Bonferroni method.

To estimate if adherence to different nutritional guidelines is associated with DSR, simple and multiple linear regression models were fitted using adherence as the predictor variable and DSR as an outcome. Only those socio-demographic variables that showed statistically significant different DSRs across categories were included in the regression models. Finally, to evaluate the association of DSR with the nutritional quality of individual diets, simple and multiple regressions were fitted using DSR as a predictor, and average NRF8·3 index scores as an outcome. Linearity and residual distributions were visually assessed, and collinearity (through tolerance level to ensure variables were not closely related) was evaluated before modelling the regressions. F-statistics were used to test the significance of each term included in the regression models. Analysis was done using R (version 4.3.3), with the following libraries: *“dplyr”, “ggplot2”, “tidyverse”, “ggpubr”, “reshape2” and “ggstatsplot”* ^(36)^.

## Results

**Figure 1.**
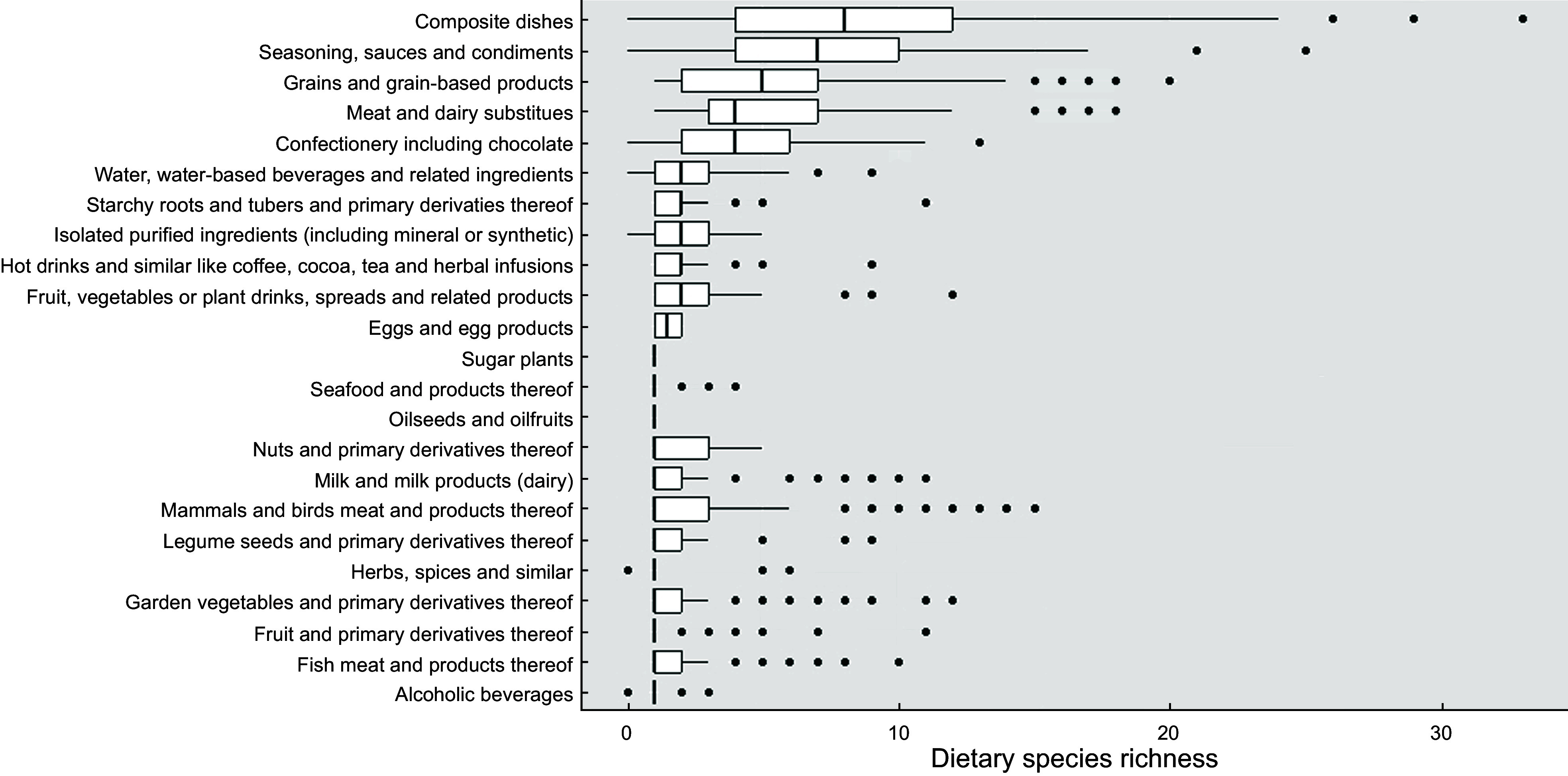
Median Dietary Species Richness for food groups including food and drinks in the NDNS nutrient databank.

Across EFSA FoodEx2 food groups, and on a per food item basis, composite dishes had the highest DSR (median of 8 (Q_1_ = 4, Q_3_ = 12)), followed by seasoning, sauces and condiments (median of 7, (Q_1_ = 4, Q_3_ = 10)) and grains and grain-based products (median of 5, (Q_1_ = 2, Q_3_ = 7)), meat and dairy substitutes (median of 4, (Q_1_ = 3, Q_3_ = 7)) and confectionery including chocolate (median DSR of 4, (Q_1_ = 2, Q_3_ = 6)). Food products in the other groups had a median DSR of 1 or 2 (Fig. 1). Within the food group of composite dishes, meat-based dishes had the highest DSR (median of 12, (Q_1_ = 7, Q_3_ = 14)), followed by legumes-based dishes (median of 10, (Q_1_ = 7, Q_3_ = 12)) and vegetable-based dishes (median of 9, (Q_1_ = 7, Q_3_ = 15)). Within the food group of seasoning, sauces and condiments, savoury sauces had the highest DSR (median of 8, (Q_1_ = 6, Q_3_ 10)). Within the food group of grains and grain-based products, cereal bars had the highest DSR (median of 10, (Q_1_ = 5, Q_3_ 12)) (see online supplementary material, Supplementary Fig. 1).

**Figure 2.**
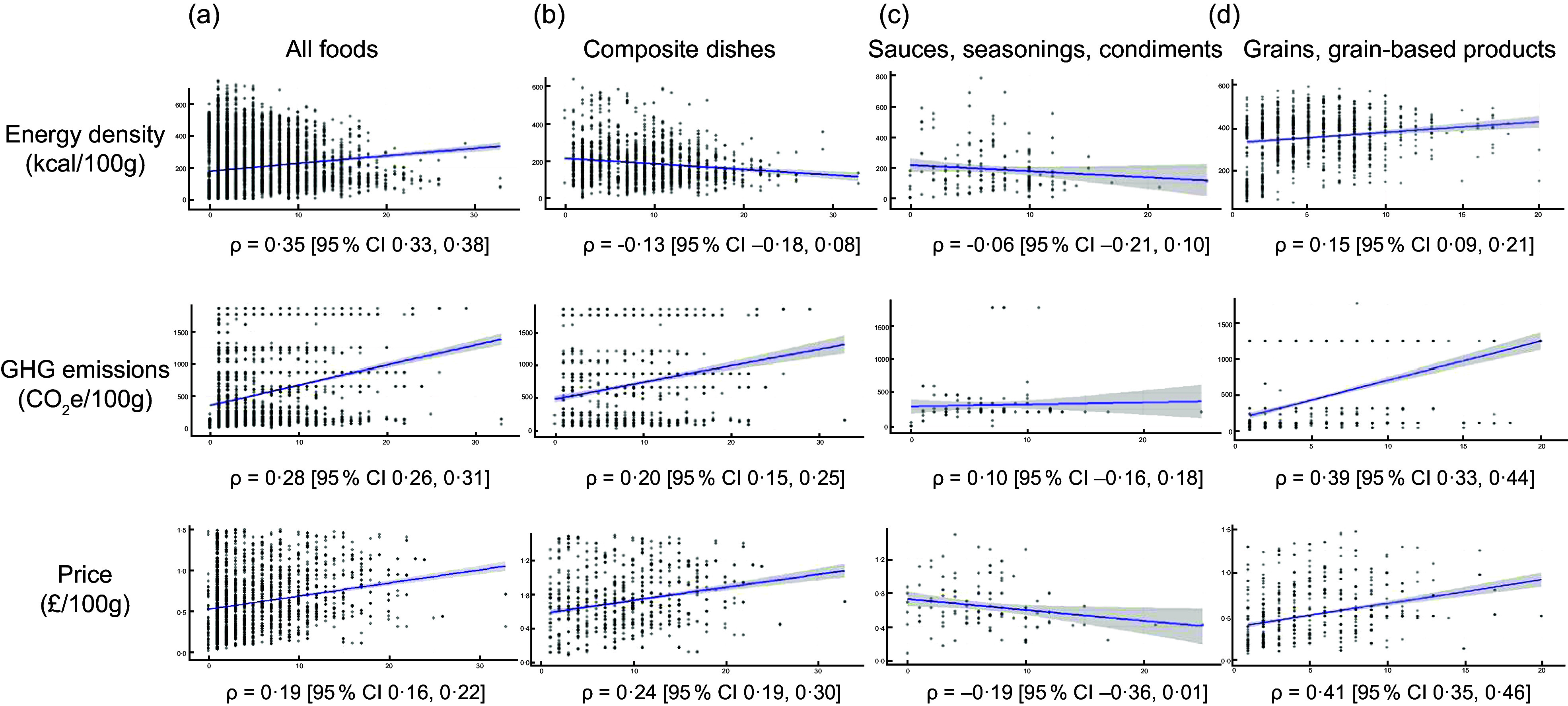
Association between Dietary Species Richness and energy density, greenhouse gas emissions and price, across foods and within the food groups composite dishes, seasoning, sauces, condiments and grains and grain-based products. All analyses were adjusted using the Bonferroni method. *ρ* = Spearman correlation coefficient; GBP = Great British Pounds price in September 2023, CO2e = gCO2-equivalents.

Across all foods, foods with a higher DSR had a higher energy density (*ρ* = 0·19, *P*-value <0·001), a higher price (*ρ* = 0·35, *P*-value <0·001) and higher GHG emissions (*ρ* = 0·28, *P*-value <0·001) (Fig. 2(a)). However, such correlations appeared food group specific. For example, within the food group of composite dishes, foods with a higher DSR had a lower energy density (*ρ* = –0·13, *P* < 0·001), higher GHG emissions (*ρ* = 0·20, *P* < 0·001) and a higher price (*ρ* = 0·24, *P* < 0·001) (Fig. 2(b)). Within the food group of sauces, seasonings and condiments, foods with a higher DSR had a lower price (–0·19, *P* = 0·03) (Fig. 2(c)). Within the food group of grains and grain based-dishes, foods with a higher DSR had a significantly higher energy density (*ρ* = 0·15, *P* < 0·001), higher GHG emissions (*ρ* = 0·39, *P* < 0·001) and a higher price (*ρ* = 0·41, *P* < 0·001) (Fig. 2(d)).

**Figure 3.**
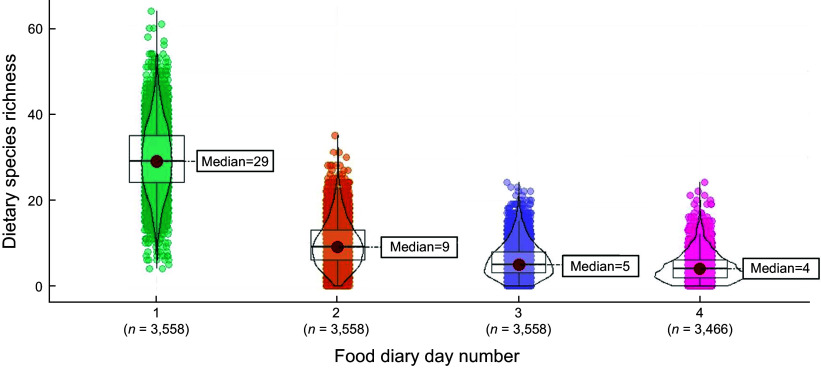
Median Dietary Species Richness for each of the four food intake assessment days.

The median DSR measured over 4 days was 49 (Q_1_ = 43, Q_3_ = 56; range 14–92). The median daily DSR, measured across 3558 participants, was 29 (Q_1_ = 24, Q_3_ = 35) for the first reporting day, with a further nine additional unique species (Q_1_ = 6, Q_3_ = 13) for the second reporting day, a further five additional unique species (Q_1_ = 3, Q_3_ = 8) for the third reporting day and a further four additional unique species (Q_1_ = 2, Q_3_ = 6) for the fourth reporting day (Fig. 3). The first 2 days covered 80 % of the median DSR measured over 4 days. Initial analysis revealed that DSR over the four reporting days significantly differed between age categories (children, adolescents, adults and elders; *P* < 0·001), household income (low, middle and high tertile; *P* < 0·001), IMD (the most deprived living, 1, to the least deprived areas, 5; *P* < 0·001) and marital status (single, married, civil partnership, separated or divorced, widow; *P* < 0·001), but not between sexes, ethnic groups (White, Mixed, Black or Black British, Asian or Asian British, other groups; *P* = 0·123), BMI categories (underweight, normal, overweight, obesity and morbid obesity; *P* = 0·382) or sex (female or male, *P* = 0·438). Differences, if any, were determined after the first day of measuring food intake (see online supplementary material, Supplementary Table 2).

When fitting a simple regression analysis, the variables, age, household income and IMD each explained a significant amount of variance in DSR, with lower age, higher household incomes and those in less deprived IMD categories predicted to have a significantly (all *P* < 0·001) higher median DSR over 4 days (Table 1). All ‘adherence to dietary recommendation’ variables also explained significant variance in median DSR over 4 days. Those consuming more than 30 g of fibre per day, more than 280 g of fish a week or at least 400 g of fruits and vegetables per day were predicted to have a significantly (all *P* < 0·001) higher median DSR over 4 days of 7, 7 and 3, respectively. Those complying with all the healthy dietary guidelines were also predicted to have a higher median DSR over 4 days of 6; however, because of low numbers (only 5 out of 3558 participants achieved this), this was not statistically significant (Table 1, see online supplementary material, Supplementary Table 3). On the other hand, the median DSR of those consuming less than 5 % of total energy from free sugars or those consuming less than 6 g of salt per day over 4 days were predicted to be 5 and 3 lower, respectively. Also, the median DSR of those consuming less than 60 % of total energy from processed or ultra-process foods was predicted to be 2 lower. In both simple and multiple regression models, age, household income, IMD and all dietary quality variables were still significant and hence included in the analysis. Overall, these predictor variables explained significant variance in median DSR over 4 days (Table 1).

**Table 1.** Regression models to identify significant sociodemographic and dietary quality predictor variables for DSR

Predictor variables	*n*	Estimate	se	*t*-value	*P*
Total number of participants	3558				
Simple linear regression models using cut-offs based on dietary guidelines					
*Sociodemographic predictor variables*					
Age (y)	–	–0·025	0·007	–3·604	<0·001
Household income (tertiles)	–	3·445	0·220	15·61	<0·001
IMD (categories)	–	1·544	0·120	12·84	<0·001
*Adherence to dietary recommendations for*					
Saturated fats	1291	–0·847	0·366	–2·337	0·019
Free sugars	416	–4·663	0·543	–8·577	<0·001
Low consumption of processed or ultra-process foods	1634	–1·836	2·551	–5·205	<0·001
Red meat	2660	0·984	0·406	2·425	0·0154
Salt	2946	–2·84	0·465	–6·11	<0·001
Fibre	1898	7·033	0·335	21·09	<0·001
Fruits and vegetables	235	6·673	0·701	9·509	<0·001
Fish	898	2·796	0·568	4·921	<0·001
Meeting all the above dietary quality variables	5	6·460	4·710	1·379	0·168
Multiple linear regression models using cut-offs based on dietary guidelines Multiple R-squared: 0·2223, adjusted R-squared: 0·2196, F-statistic: 79·61 on 11 and 3063 DF, *P* < 0·001					
*Socio-demographic predictor variables*					
Age	–	–0·064	0·007	–8·639	<0·001
Household Income	–	2·24	0·219	10·201	<0·001
IMD	–	0·781	0·125	6·242	<0·001
*Adherence to dietary recommendations for*					
Saturated fats	1291	–1·483	0·353	–4·200	<0·001
Free sugars	416	–4·432	0·531	–8·347	<0·001
Low consumption of processed or ultra-process foods	1634	–0·967	0·336	–2·877	<0·001
Red meat	2660	1·478	0·403	3·661	<0·001
Salt	2946	–1·203	0·477	–2·522	0·011
Fibre	1898	5·737	0·372	15·410	<0·001
Fruits and vegetables	235	5·013	0·706	7·096	<0·001
Fish	898	1·753	0·560	3·129	0·001

Four-day dietary data was used to perform the regression analysis. People categorised as IMD 1 represent 20 % of the most deprived, while those categorised as IMD 5 are 20 % of the least deprived. Household income was categorised into tertiles; those in the first tertile group had the lowest income, and those in the third tertile group had the highest incomes. Cut-offs for dietary quality predictors follow national dietary guidelines; see the methods section for more details.

When fitting regression analysis to evaluate the association of DSR with the nutritional quality (measured through NRF 8·3 index scores per 100 kcal) of individual diets, we found that DSR could predict a small but significant increase in NRF8·3 index scores per 100 kcal (estimate 168·9, se 8·01, *t*-value 21·07, *P* < 0·001), which remained significant after adjusting for age, household income and IMD (estimate 159·3, se 8·71, *t*-value 18·13, *P* < 0·001) (see online supplementary material, Supplementary Fig. 2). This means that with every unit increase in DSR, the average NRF8·3 index score per 100 kcal would increase to a small extent, showcasing a better nutritional quality.

## Discussion

Here, we show that DSR can be robustly measured using NDNS 4-day food intake data and that DSR can be used as an indicator of food biodiversity in UK diets. We identified 216 relevant, unique species across foods, food groups and diets. Median DSR was 49 over 4 days, with 80 % of the unique species consumed over 4 days being captured in the first 2 days of recall. DSR was significantly higher in those who were younger, those who had a higher household income and those residing in the least deprived areas, but DSR did not differ between sex, ethnic groups or BMI categories. Composite dishes, especially meat-based dishes, mainly contributed to DSR in UK diets, followed by seasoning, sauces and condiments and grains and grain-based products. Adherence to different dietary guidelines, especially in relation to fibre, fruits and vegetables and fish consumption, was also associated with a significantly higher DSR. However, those with a lower consumption of dietary saturated fats, free sugars and processed or ultra-processed foods were predicted to have a significantly lower DSR. A higher DSR was generally associated with a higher diet quality, but the effect estimate was small.

Food biodiversity is a relatively novel concept, and there is currently no standardised methodology to calculate DSR. Most studies assessing DSR have been conducted in low- and middle-income countries. These studies used 24-hour diet recalls or ecological assessments to identify DSR across diets (ranging from 40 to 234), with DSR or animal protein species richness ranging from 8 to 24, or from 44 to 52, respectively, depending on whether DSR or animal protein species richness was assessed during the dry or wet season in some of the studies^(12,37,38)^. An analysis of DSR in the EPIC cohort found a median DSR of 68 per person per year, calculated from FFQ, out of a list of 248 unique species^(5)^. In our study, using NDNS survey data, we found a median DSR of 49 from four daily food diaries on consecutive days, including weekdays and a weekend day. Whilst DSR would have increased with more recording days, we found that 80 % of the unique species consumed were reported within the first 2 days of recording food intake, suggesting that a 2-day food diary captured the majority of species diversity when calculating DSR from 4-day NDNS food diaries. However, more days over different periods may be required for low- and middle-income countries where dietary diversity depends more on seasons^(12,37,38)^. The higher value for DSR in the study by Hanley-Cook^(5)^, compared with our study, may reflect a much wider geographical sampling area, and the FFQ covering a much longer period of dietary intake which would have captured a higher number of less frequently consumed food items. On the other hand, that study was based on an observational cohort using FFQ, and exposure misclassification and residual confounding cannot be ruled out^(5)^.

It is essential to understand how food biodiversity is associated with dietary quality and health outcomes to justify its use as a meaningful new metric that can link diets to human and planetary health, complementing existing indicators for healthy and sustainable diets. Associations between dietary diversity indicators and health outcomes, such as body weight and non-communicable diseases, have been largely inconsistent^(2)^. However, Hanley-Cook^(5)^ showed, in the largest study of its kind, that DSR was inversely associated with total mortality and mortality due to cancer, heart disease, digestive disease and respiratory disease, independent of socio-demographic, lifestyle and other known dietary risk factors such as intake of energy, meat and fibre. This suggests that DSR may provide health benefits beyond dietary quality alone. Absolute death rates among participants in the highest and lowest fifth of DSR were 65·4 and 69·3 cases/10,000 person-years, respectively (hazard ratio and 95 % CI: 0·63 (0·59, 0·66)), providing a powerful association between low DSR and disease outcomes across nine European countries^(5)^. In another study, DSR was linked to a higher diet quality (e.g. mean adequacies of vitamin A, vitamin C, folate, Ca, Fe and Zn) and a higher diet diversity score in women and young children in rural areas from seven low- and middle-income countries, in both the wet and the dry season^(12)^. Our study explored the link between DSR and dietary quality using the nationally representative UK NDNS database. We found that DSR was driven by composite dishes on the food level. Furthermore, we found that those consuming at least 30 g of fibre per day, at least 400 g of fruits and vegetables per day or more than 280 g of fish a week, all representing important dietary guidelines, were predicted to have a significantly higher DSR. The highest proportion of species was provided by different fish, fish products and any other marine and freshwater food products (29 %), fruit (24 %) and vegetable species (22 %). These categories also showed the largest amount of species diversity, indicating opportunities for increasing DSR in existing products through reformulation.

Increasing the DSR of our diets would arguably improve human health and benefit the environment, linking in-farm and on-plate biodiversity^(3)^ and serving an ecological as well as societal role. Moreover, diversifying consumption of crops, fruits, livestock and aquatic species would strengthen nutrition security. This is particularly important in low- and middle-income countries, where higher agricultural biodiversity has been associated with more diverse diets through subsistence and income-generating pathways. Greater crop species richness has been associated with small but positive increments in child height for age outcomes^(11)^. In our study, a higher DSR was associated with a higher value for the NRF8·3 index score, but the estimate for change in diet quality was relatively small. In contrast, adhering to current dietary guidelines for fruits and vegetables and fish consumption indicated an effective approach to increasing DSR. Together, this implies that the number of species alone does not necessarily predict dietary quality, but that the level of intake of unique species from ‘healthier’ food groups, such as fruit and vegetables and fish, as stipulated in the dietary guidelines, also plays an important role. This is an important finding, especially in the UK, where adherence to dietary guidelines is low – only 26 % and 17 % of the UK population adhere to recommendations for fruits and vegetables and oily fish, respectively. Intermediate to high adherence to Eat Well Guide recommendations, especially those for fruit and vegetable consumption, has been associated with a 10 % reduction in the risk of mortality and a lower carbon footprint^(39)^.

Recently, it was established that aquatic species richness in the ocean is critical for the ecosystem’s multifunctionality and provides significant nutritional benefits in relation to recommended nutrient intakes for humans, as nutrient concentrations vary substantially across aquatic food species^(40)^. Interestingly, the benefits of aquatic species richness for human consumption exceeded the diversity effects of plant and forest species richness^(40)^. The diversity of the UK fruit and vegetable supply has increased significantly in the past decades, with an increased contribution of tropical fruits, but a declining contribution of more traditional vegetables, such as cabbages and carrots^(41)^. Currently, most of the fruits, vegetables and fish we consume in the UK are imported, but the UK’s fruit and vegetable supply is increasingly dependent on imports from climate-vulnerable producing countries^(41,42)^. For instance, it has been reported that in the UK only 7 % of fruits are produced domestically, with the rest imported, largely (70 %) from outside of Europe^(42)^. In addition, it is estimated that meeting the recommendations for fruit and vegetables, or oily fish, is 16–17 % more expensive than the costs of an average 2000 kcal diet in the UK^(43)^. Therefore, to achieve impact, higher DSR foods and diets must not only be within planetary boundaries, but also affordable^(44)^. Indeed, our analysis showed that household income and the deprivation level of participants were strong and significant predictors of DSR in the UK diet, with those with higher incomes and least deprived having a significantly higher DSR. To increase DSR in UK diets in a just manner, we will need to ensure that we conserve natural biodiversity worldwide through our dietary choices and maintain the affordability of high-DSR foods and diets, which requires integration of environmental and public health policies^(45,46)^.

The most important strength of this study is the robust bottom-up analysis of DSR in UK diets, using 4-day food intake data collected from 3558 participants across sex, age, ethnicity and socio-economic groups over a recent period of 3 years. Another strength is using an in-house NDNS nutrient databank, allowing us to link nutrient composition, FoodEx2 food classification, ingredients, number and identity of unique species, GHG emissions and cost to calculate dietary quality indicators and DSR on the food and diet level. In this study, we included unique species from all foods apart from extracts and flavourings with unknown or unfamiliar nomenclature. There may be reasons to exclude certain foods and/or ingredients such as herbs, spices, flavourings and extracts, but including all foods *v*. excluding the lowest 5 % or 10 % species intake from each of the food groups did not substantially change the protective effect of DSR on all-cause mortality^(5)^. Limitations include our current inability to link DSR to important health outcomes using the NDNS database and the inability to measure DSR over a period of more than 4 days, which would likely have resulted in higher DSR values. Also, DSR does not assess ‘evenness’, which is a key component of diversity. Therefore, DSR gives rare edible species the same weight as more common species, in kcal/day. Finally, we did not correct for potential under-overreporting, as the outcome (DSR) relates to the number of unique species consumed rather than its amount. Underreporting may have led to fewer foods being reported and, therefore, to a lower DSR, which means that real DSR values may have been underestimated for some participants.

In conclusion, we established DSR in UK diets, based on four-day food intake data, as a case study for countries that have a relatively high consumption of processed foods as part of Western-style diets. We found that DSR is significantly higher in those adhering to dietary guidelines for fruit and vegetables, fish and fibre intake. DSR is also higher in younger people, those with higher household incomes and those living in the least deprived areas. Composite dishes contributed mostly to DSR and, therefore, offer an opportunity to increase DSR through food reformulation, although our data show the importance of the level of intake of unique species from ‘healthier’ food groups, such as fruit and vegetables and fish, as already set out in the dietary guidelines.

## Supporting information

Aceves-Martins et al. supplementary material Aceves-Martins et al. supplementary material

## Supplementary material

For supplementary material accompanying this paper visit https://doi.org/10.1017/S1368980025000473
