# Supplementary material for: The measurement of dietary species richness reveals that a higher consumption of dietary fibre, fish, fruits and vegetables, is associated with greater food biodiversity in UK diets
Source: Public Health Nutr. 2025 Apr 11;28(1):e76. doi: 10.1017/S1368980025000473 (PMC12100553; doi:10.1017/S1368980025000473)

**Supplementary Data**

**Supplementary Table 1.** List of 269 unique species identified.

| **Abalone** |
| --- |
| **Akee apples** |
| **Alfalfa** |
| **Alfonsino** |
| **Almond, Almonds** |
| **Amberjack** |
| **Anchovies** |
| **Anise seed** |
| **Apples** |
| **Apricots** |
| **Arctic char** |
| **Asian rice** |
| **Asparagus** |
| **Atlantic halibut** |
| **Atlantic mackerel** |
| **Atlantic pomfret** |
| **Atlantic salmon** |
| **Aubergine** |
| **Avocado** |
| **Bamboo** |
| **Barley** |
| **Barnacle** |
| **Basil** |
| **Beets, chards** |
| **Black eyed peas** |
| **Blackberry** |
| **Blackcurrant** |
| **Blue shark** |
| **Blue whiting** |
| **Blueberries** |
| **Bonito** |
| **Borage** |
| **Brazil nuts** |
| **Breadfruits** |
| **Broad beans** |
| **Buckwheat** |
| **Buffalo** |
| **Burr gherkin** |
| **Butternut squashes** |
| **Button mushrooms** |
| **Caper** |
| **Cardamon Fruit** |
| **Carobs** |
| **Carrots** |
| **Cashew nut** |
| **Cassava** |
| **Celeriac, celery** |
| **Chayote fruits** |
| **Cherimoya** |
| **Chestnuts** |
| **Chicken** |
| **Chickling vetches** |
| **Chickpea** |
| **Chili peppers** |
| **Chinese cabbages, turnip** |
| **Chive** |
| **Cinnamon bark** |
| **Cloudberry** |
| **Coalfish** |
| **Cockles** |
| **Cocoa bean** |
| **Coconut** |
| **Cod** |
| **Coffee bean** |
| **Common banana, plantain** |
| **Common beans** |
| **Common dab** |
| **Common mussel** |
| **Common nettle** |
| **Common peaches** |
| **Common periwinkle** |
| **Common shrimps** |
| **Common skate** |
| **Common wheat, spelt** |
| **Coriander leaves** |
| **Courgette, pumpkin, squash** |
| **Cow** |
| **Cowberries, lingonberries** |
| **Cranberries** |
| **Cucumber, gherkin** |
| **Cumin Seeds** |
| **Curly endives** |
| **Cuttlefishes** |
| **Date** |
| **Dill** |
| **Duck** |
| **Durum wheat** |
| **Edible crab** |
| **European conger** |
| **European eel** |
| **European freshwater bream** |
| **European freshwater crayfish** |
| **European moose** |
| **European oyster** |
| **European perch** |
| **European plaice** |
| **European sardine** |
| **European spider crab** |
| **European sprat** |
| **Fennel** |
| **Fig** |
| **Flounders** |
| **Gages** |
| **Garden snail** |
| **Garfish** |
| **Garlic** |
| **Giant tiger prawn** |
| **Gilthead seabream** |
| **Ginger** |
| **Globe artichoke, cardoon** |
| **Goat** |
| **Gojiberry** |
| **Goose** |
| **Gooseberry** |
| **Grapefruits** |
| **Grapes** |
| **Groupers** |
| **Guava** |
| **Haddock** |
| **Hakes** |
| **Hare** |
| **Hazelnut** |
| **Head cabbages** |
| **Head lettuces** |
| **Hemp seeds** |
| **Herrings** |
| **Hibiscus infusion flowers** |
| **Hijiki** |
| **Horse** |
| **Horse mackerel** |
| **Horseradish root** |
| **Jerusalem artichoke** |
| **Kaki** |
| **Kiwi** |
| **Kumquats** |
| **Leek** |
| **Lemon** |
| **Lentil** |
| **Lime** |
| **Ling** |
| **Linseed** |
| **Litchis** |
| **Loquats** |
| **Lumpfish** |
| **Macadamias** |
| **Maize** |
| **Mandarins, clementine** |
| **Mango** |
| **Marble goby** |
| **Marjoram Fruit** |
| **Medlar** |
| **Megrims** |
| **Melons** |
| **Mints** |
| **Monkfish, anglerfish** |
| **Mulberries (black and white)** |
| **Mullets** |
| **Mung beans** |
| **Mustard seeds** |
| **Nectarines** |
| **Northern pike** |
| **Northern prawn** |
| **Norway pout** |
| **Norwegian lobster** |
| **Nutmeg seed** |
| **Oat** |
| **Octopuses** |
| **Oil palms** |
| **Okra** |
| **Olive** |
| **Onions** |
| **Oranges** |
| **Oregano** |
| **Pangas catfishes** |
| **Papayas** |
| **Parsley** |
| **Parsnip root** |
| **Partridge fresh meat** |
| **Passionfruit** |
| **Peanut** |
| **Pear** |
| **Pearl millet** |
| **Peas** |
| **Pecan** |
| **Peppercorn** |
| **Pheasant** |
| **Pig, boar** |
| **Pigeon** |
| **Pike-perch** |
| **Pine nuts** |
| **Pineapple** |
| **Pistachio** |
| **Plum** |
| **Pollock** |
| **Pomegranate** |
| **Poppy seeds** |
| **Potato** |
| **Prickly pear** |
| **Ptarmigan** |
| **Purple urchin** |
| **Quail** |
| **Quinces** |
| **Quinoa grain** |
| **Rabbit** |
| **Radicchio, Belgian endives** |
| **Radish, daikon** |
| **Raspberries** |
| **Razor clam** |
| **Red mustard leaves** |
| **Redcurrant** |
| **Rhubarb** |
| **Rockweed** |
| **Roe deer** |
| **Roman rocket and similar-** |
| **Rose hips** |
| **Rosemary** |
| **Rye** |
| **Safflower seeds** |
| **Saffron** |
| **Sage** |
| **Sago plant** |
| **Salsify leaves** |
| **Sapodillas** |
| **Scallops, pectens** |
| **Scorpion fish** |
| **Sea bass** |
| **Sea bream** |
| **Sea catfish** |
| **Sea lettuce** |
| **Sesame** |
| **Shea nuts** |
| **Sheep** |
| **Smelt** |
| **Smooth hounds** |
| **Snappers** |
| **Sole** |
| **Soybean** |
| **Spinach** |
| **Spiny dogfish** |
| **Squids** |
| **Strawberry** |
| **Sturgeon** |
| **Sunflower** |
| **Swede, rapeseed** |
| **Sweet cherry** |
| **Sweet corn** |
| **Sweet pepper** |
| **Sweet potato** |
| **Swordfish** |
| **Tamarind** |
| **Tarragon** |
| **Tea leaves** |
| **Thyme** |
| **Tomatoes** |
| **Trout** |
| **Tuna** |
| **Turbot** |
| **Turkey** |
| **Walnut** |
| **Watercress** |
| **Watermelon** |
| **Whelks** |
| **White lupine** |
| **Whitefish, Coregonus** |
| **Whiting** |
| **Witloofs** |
| **Wolffish** |
| **Yam** |
| **Yeast cultures** |

**Those species highlighted in orange were identified in the EPIC cohort's food list (See Hanley-Cook et al. 2021) but were not identified across the NDNS food and drink products.*

**Supplementary Figure 1.** Median Dietary Species Richness (DSR) across composite dishes (A), seasoning, sauces, and condiments (B), and grains and grain-based products (C).

***
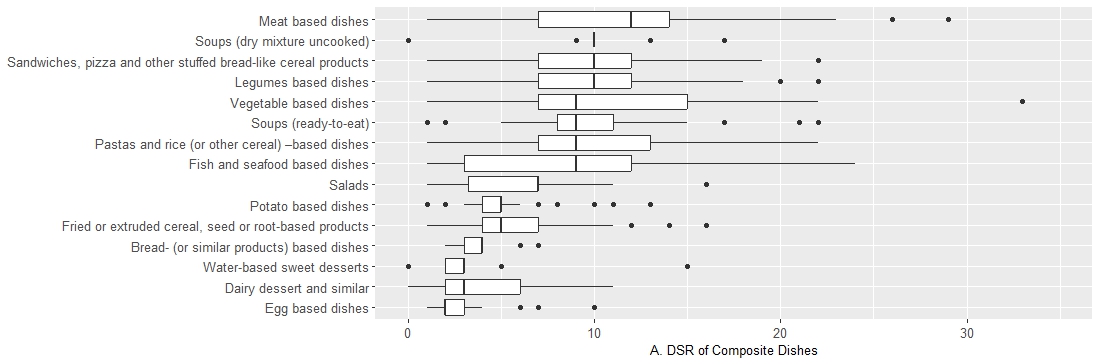
***


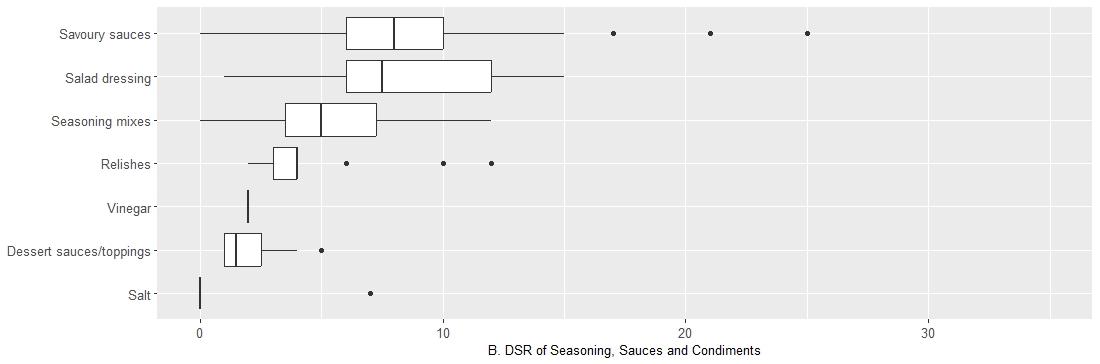


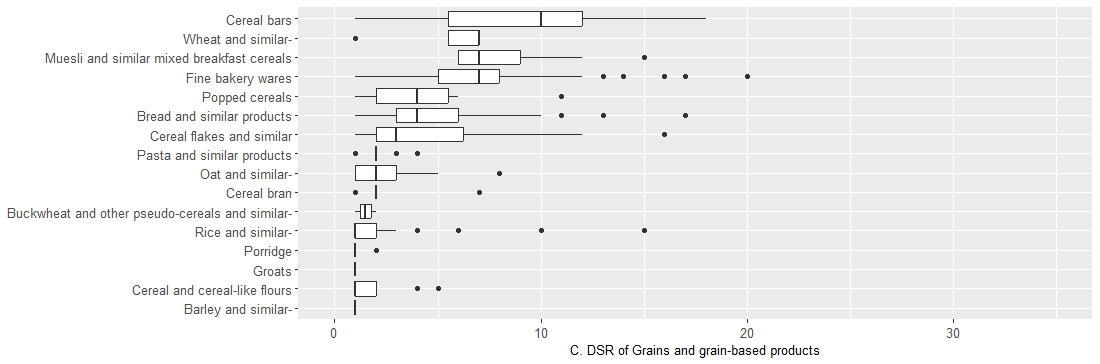


**Supplementary Table 2**. Differences in median Dietary Species Richness (DSR) across sociodemographic characteristics.

|  | | **Median DSR (IQR)** | | | |  |
| --- | --- | --- | --- | --- | --- | --- |
|  |  | **Day** | | | |  |
|  |  | **1** | **2** | **3** | **4** |  |
| **Sex** | Women | 29.5 (12) | 9 (7) | 6 (6) | 4 (4) | 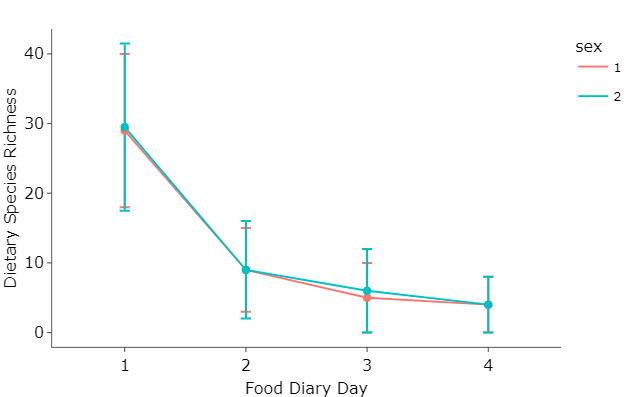 |
|  | Men | 29 (11) | 9 (6) | 5 (5) | 4 (4) |  |
|  |  | *p=0.438* |  | | |  |
| **Age** | Children | **31 (10)** | 9 (6) | 5 (5) | 4 (4) | 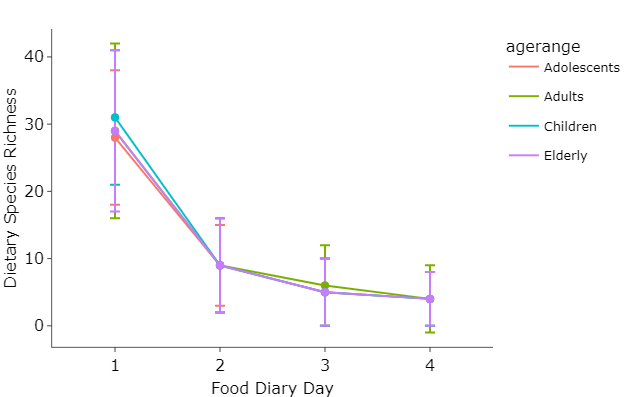 |
|  | Adolescents | **28 (10)** | 9 (7) | 6 (6) | 4 (4) |  |
|  | Adults | **29 (13)** | 9 (7) | 5 (5) | 4 (5) |  |
|  | Elders | **29 (12)** | 9 (7) | 5 (5) | 4 (4) |  |
|  |  | ***p <0.001*** |  | | |  |
| **Household income** | Lowest income tertile | **27 (10.5)** | 8 (7) | 5 (5) | 4 (4) | 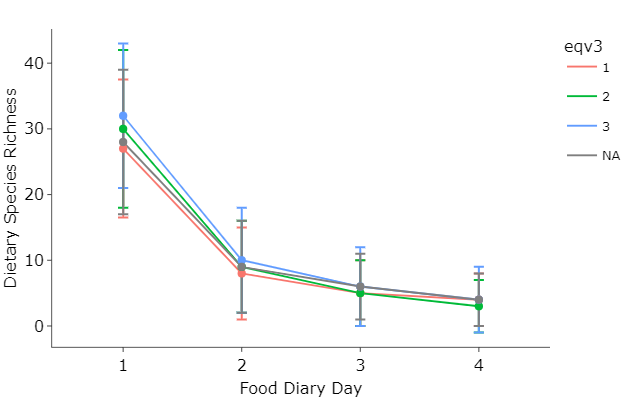 |
|  | Middle income tertile | **30 (12)** | 9 (7) | 5 (5) | 3 (4) |  |
|  | Highest income tertile | **32 (11)** | 10 (8) | 6 (6) | 4 (5) |  |
|  | NA | **28 (10.5)** | 9 (7) | 5 (5) | 4 (4) |  |
|  |  | ***P <0.001*** |  | | |  |
| **IMD** | 1 | **28 (11)** | 8 (7) | 5 (5) | 3 (4) | 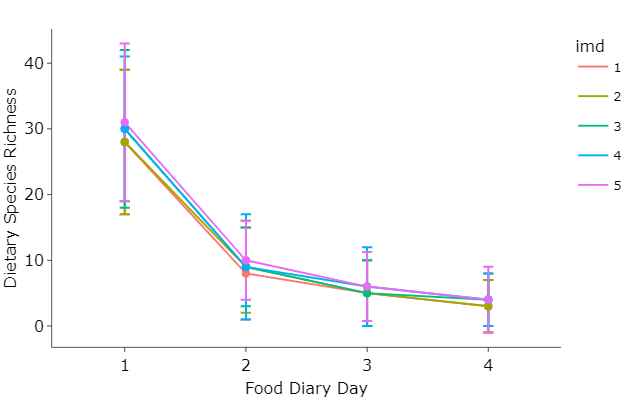 |
|  | 2 | **28 (12)** | 9 (7) | 5 (5) | 3 (4) |  |
|  | 3 | **30 (11)** | 9 (6) | 5 (5) | 4 (4) |  |
|  | 4 | **30 (11)** | 9 (8) | 6 (6) | 4 (4) |  |
|  | 5 | **31 (12)** | 10 (6) | 6 (5.5) | 4 (5) |  |
|  |  | ***P <0.001*** |  | | |  |
| **Ethnic group** | White | 29 (11) | 9 (7) | 5 (5) | 4 (4) | 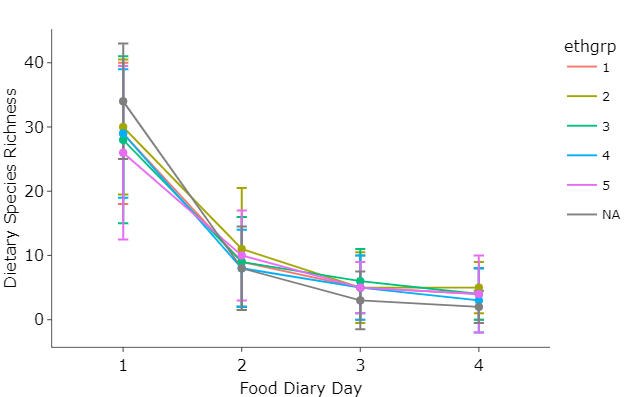 |
|  | Mixed ethnic group | 30 (10.5) | 11 (9.5) | 5 (5.5) | 5 (4) |  |
|  | Black or Black British | 28 (13) | 9 (7) | 5 (5) | 4 (4) |  |
|  | Asian or Asian British | 29 (10) | 8 (6) | 6 (6) | 3 (5) |  |
|  | Any other group | 26 (13.5) | 10 (7) | 5 (4) | 4 (6) |  |
|  | NA | 34 (10) | 8 (5) | 3 (4) | 2 (3) |  |
|  |  | *p=0.123* |  |  |  |  |
| **Marital status** | Single | **28 (12)** | 9 (7) | 5 (5) | 4 (4) | 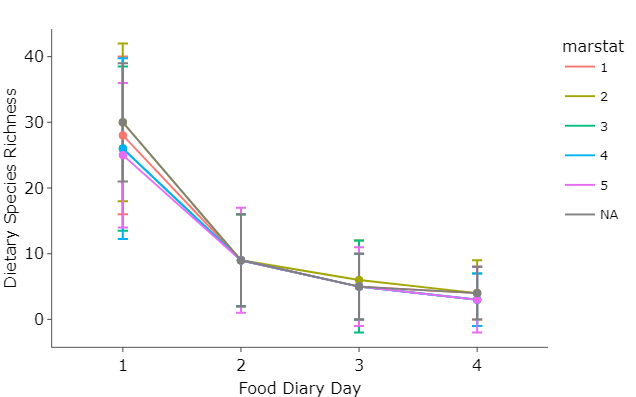 |
|  | Married | **30 (12)** | 9 (7) | 6 (6) | 4 (5) |  |
|  | Civil partnership | **26 (12.5)** | 9 (7) | 5 (7) | 3 (4) |  |
|  | Separated/  divorced | **26 (13.75)** | 9 (7) | 5 (5) | 3 (4) |  |
|  | Widow | **25 (11)** | 9 (8) | 5 (6) | 3 (5) |  |
|  | NA | **30 (12)** | 9 (7) | 5 (7) | 3 (4) |  |
|  |  | ***p<0.001*** |  | | |  |
| **BMI** | Underweight | 30 (11) | 7 (10) | 5 (5) | 3 (5) | 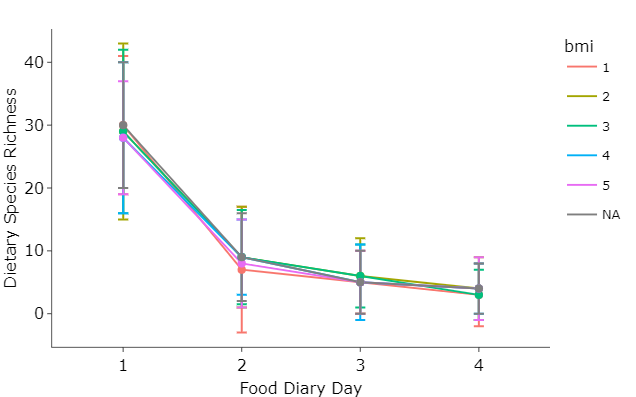 |
|  | Normal weight | 29 (14) | 9 (8) | 6 (6) | 4 (5) |  |
|  | Overweight | 29 (13) | 9 (7.5) | 6 (5) | 3 (4) |  |
|  | Obesity | 28 (12) | 9 (6) | 5 (6) | 4 (4) |  |
|  | Morbid Obesity | 28 (9) | 8 (7) | 5 (5) | 4 (5) |  |
|  | NA | 30 (11) | 9 (10) | 5 (5) | 4 (5) |  |
|  |  | *p=0.382* |  | | |  |

**Supplementary Table 3.** Differences in median Dietary Species Richness (DSR) across dietary quality categories

| **Variables** | | **Median DSR (IQR)** | | | | | **Graph** |
| --- | --- | --- | --- | --- | --- | --- | --- |
|  |  | **Day** | | | | |  |
|  |  | **1** | | **2** | **3** | **4** |  |
| **Saturated fat consumption** | Consuming more than 11% of total energy from saturated fats | 30 (11) | | 9 (7) | 5 (5) | 4 (4) | 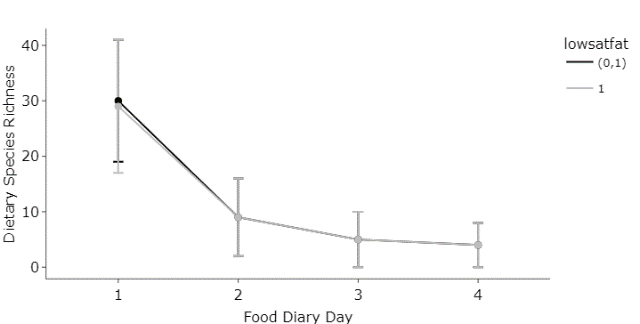 |
|  | Consuming less than 11% of total energy from saturated fats | 29 (12) | | 9 (7) | 5 (5) | 4 (4) |  |
|  |  | *p=0.151* | |  | | |  |
| **Free sugar consumption** | Consuming more than 5% of total energy from free sugars | **30 (12)** | | 9 (7) | 6 (5) | 4 (4) | 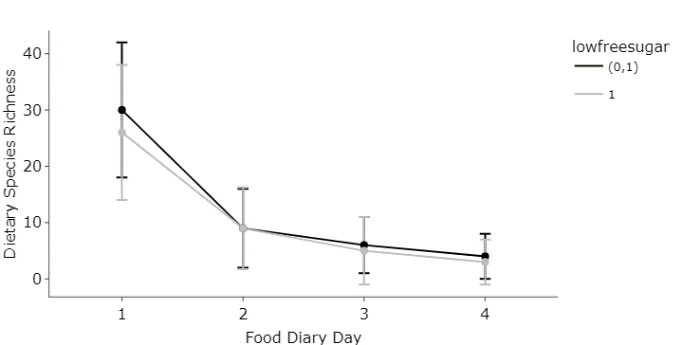 |
|  | Consuming less than 5% of total energy from free sugars | **26 (12)** | | 9 (7) | 5 (6) | 3 (4) |  |
|  |  | ***p<0.000*** | |  | | |  |
| **Processed and ultra-processed food consumption** | Consuming more than 60% of total energy from processed and ultra-processed food | **30 (11)** | | 9 (7) | 6 (5) | 4 (4) | 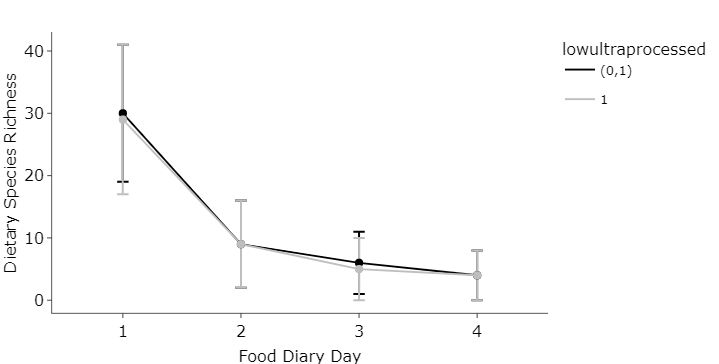 |
|  | Consuming less than 60% of total energy from processed and ultra-processed food | **29 (12)** | | 9 (7) | 5 (5) | 4 (4) |  |
|  |  | ***p<0.002*** | |  | | |  |
| **Red meat consumption** | Consuming more than 280g of red meat across four days. | **29 (11)** | | 9 (7) | 5 (5) | 4 (4) | 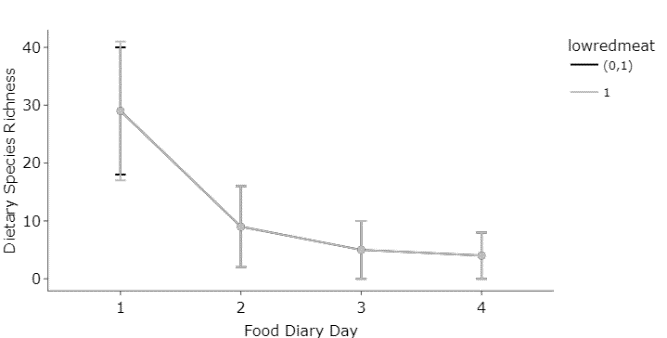 |
|  | Consuming less than 280g of red meat across four days. | **29 (12)** | | 9 (7) | 5 (5) | 4 (4) |  |
|  |  | *p=0.363* | |  |  |  |  |
| **Salt consumption** | Consuming more than 24g of salt across four days. | **32 (11)** | | 9 (7) | 6 (5) | 4 (4) | 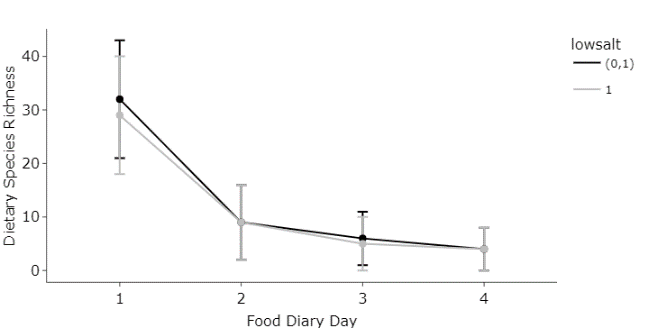 |
|  | Consuming less than 24g of salt across four days. | **29 (11)** | | 9 (7) | 5 (5) | 4 (4) |  |
|  |  | ***p<0.000*** | |  | | |  |
| **Fibre consumption** | Consuming less than 120 g of fibre across four days. | **27 (10)** | | 9 (7) | 5 (5) | 4 (4) | 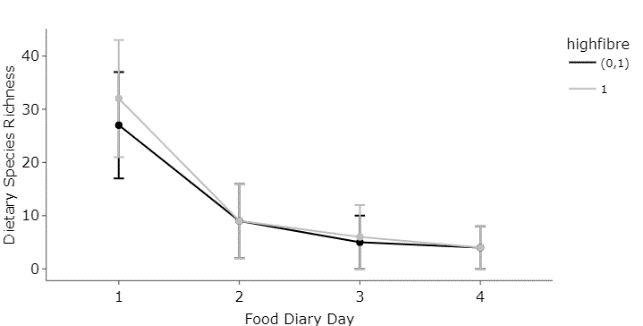 |
|  | Consuming more than 120 g of fibre across four days. | **32 (11)** | | 9 (7) | 6 (6) | 4 (4) |  |
|  |  | ***p<0.000*** | |  | | |  |
| **Fruit and vegetable consumption** | Consuming less than 1600 g of fruit and vegetables across four days. | **29 (11)** | | 9 (7) | 5 (5) | 4 (4) | 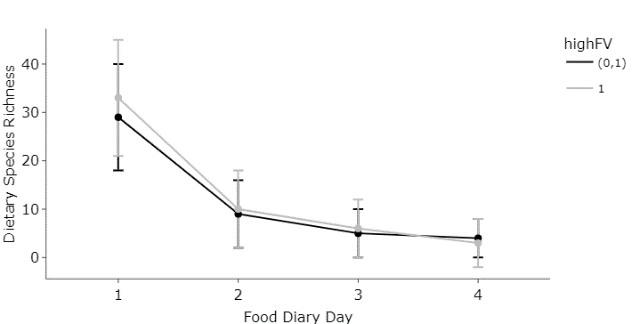 |
|  | Consuming more than 1600 g of fruit and vegetables across four days. | **33 (12)** | | 10 (8) | 6 (6) | 3 (5) |  |
|  |  | ***p<0.000*** | |  | | |  |
| **Fish consumption** | Consuming less than 160g of fish across four days. | **29 (11)** | | 9 (7) | 5 (5) | 4 (4) | 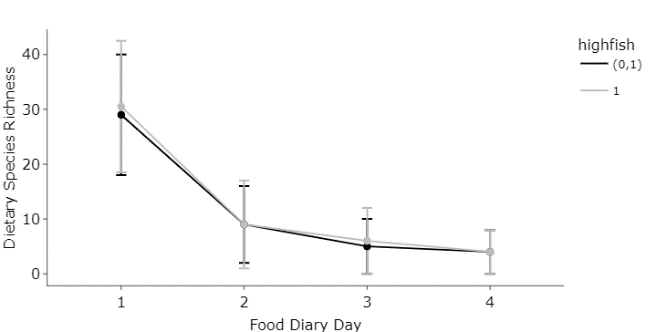 |
|  | Consuming more than 160g of fish across four days. | **30.5 (12)** | | 9 (8) | 6 (6) | 4 (4) |  |
|  |  | | ***p=0.049*** |  | | |  |
| **Following all the above-enlisted guidelines.** | Not meeting one or more guidelines. | **29 (11)** | | 9 (7) | 5 (5) | 4 (4) | 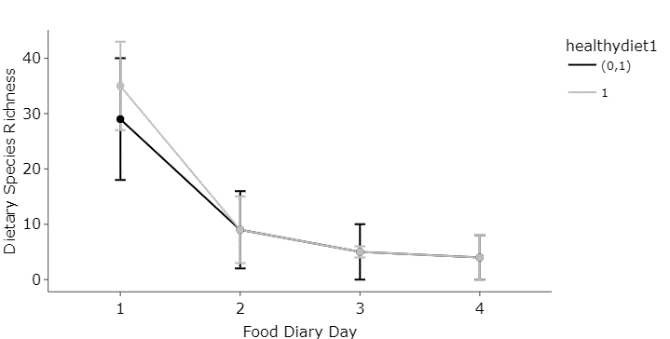 |
|  | Meeting all guidelines. | **35 (8)** | | 9 (6) | 5 (5) | 4 (5) |  |
|  |  | | *p=*0.288 |  | | |  |

**Supplementary Figure 2.** Regression analysis to evaluate the association of DSR with the nutritional quality (measured through NRF 8.3 as a proxy) of individual diets


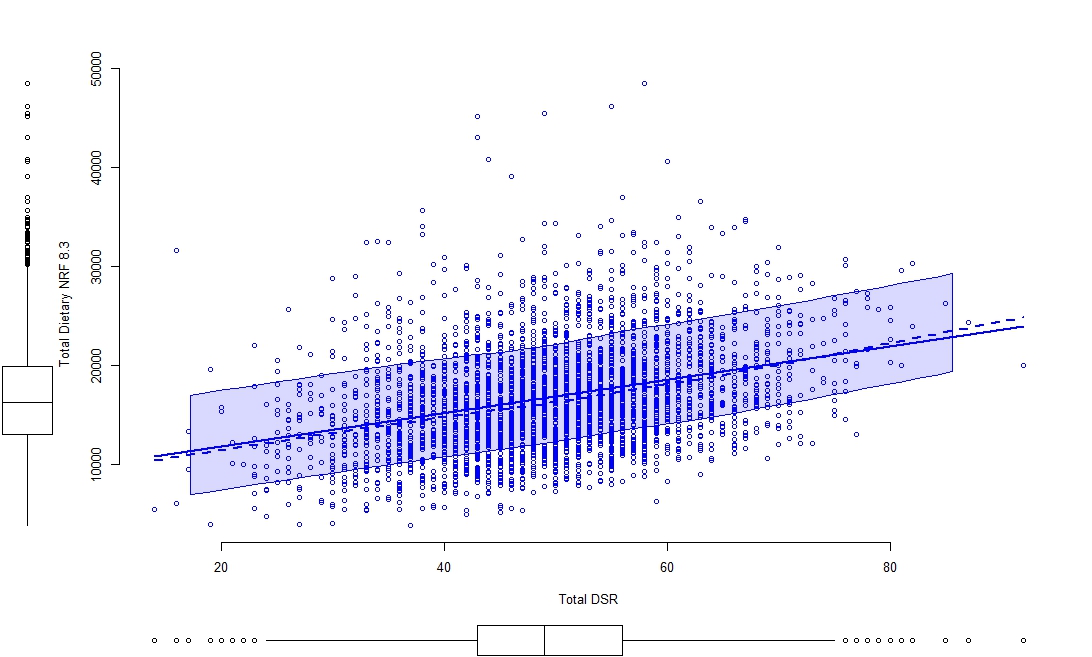

Supplement: Aceves-Martins et al. supplementary material [file S1368980025000473sup001.docx]
